# Regulating Glycerol Metabolism to Investigate the Effects of Engineered *Saccharomyces cerevisiae* on Simulated Wine Flavor Compounds

**DOI:** 10.3390/foods15020300

**Published:** 2026-01-14

**Authors:** Lu Chen, Junjie Gao, Huiyan Wang, Guantong Liu, Huimin Yang, Yi Qin

**Affiliations:** 1 College of Enology, Northwest A&F University, Yangling 712100, China; 2 Xinjiang Research Institute of Agriculture in Arid Areas, Urumqi 830091, China

**Keywords:** *Saccharomyces cerevisiae*, glycerol metabolism, alcohols, esters, transcriptome

## Abstract

This study aimed to modify metabolite synthesis in *Saccharomyces cerevisiae* (*S. cerevisiae*) under simulated wine fermentation conditions by regulating the glycerol metabolic pathway. We systematically analyzed the effects of overexpressing the aquaporin gene *AQY1* and co-expressing *AQY1* with the glycerol-3-phosphate dehydrogenase gene *GPD1* on the metabolism of ethanol, higher alcohols, and esters. Our results indicate that *AQY1* overexpression increased glycerol yield by 6.58%, reduced higher alcohol content by 14.60%, and elevated ester content by 7.15%. The downregulation of related amino acid metabolism genes correlated with the observed decrease in higher alcohol levels. Notably, co-expression of *AQY1* and *GPD1* further enhanced glycerol yield by 10.66% while decreasing ethanol content by 6.32%. By analyzing changes in gene expression alongside metabolic mechanisms, we hypothesize that the redistribution of carbon flux and NADH toward the glycerol pathway not only decreases the precursors for ethanol synthesis but also directly inhibits the activity of aldehyde dehydrogenase (*ALD2*/*3*/*4*/*6*), thereby constraining ethanol production. In comparison to *AQY1* overexpression alone, the co-expression strategy did not significantly alter glycerol accumulation; however, it reduced both ethanol and ester content by 8.38% and 8.40%, respectively, while markedly increasing higher alcohol content by 22.30%. This increase may result from enhanced glycolytic flux and pyruvate accumulation, which promote metabolic flow toward amino acid synthesis pathways. In summary, this study effectively remodeled the central carbon metabolism network by targeting glycerol metabolism, achieving diverse metabolic product synthesis and providing important references for the selection and breeding of industrial *S. cerevisiae* strains.

## 1. Introduction

The balance of wine arises from the intricate interplay among its sweetness, acidity, bitterness, and aromatic compounds. The primary alcoholic components found in wine include glycerol, ethanol, and higher alcohols [1,2]. Glycerol, a key byproduct of alcoholic fermentation, enhances flavor intensity, improves mouthfeel, adds complexity to the aroma profile, and contributes to a smooth texture [3,4]. Ethanol serves as an aroma carrier; however, when present in excessive amounts, it can introduce bitterness and mask fruitiness, resulting in sensory imbalance [5,6,7]. Higher alcohols, specifically mono-hydroxy alcohols with three or more carbon atoms, rank among the most abundant flavor compounds in wine. In moderate quantities, they contribute to body and rich aromas, but when excessive, they can produce pungent sensations and bitterness, impairing both flavor and texture, and may even lead to headaches or nausea [8,9]. Aromatic compounds, particularly esters formed through acid–alcohol reactions during fermentation, are also crucial to wine’s sensory profile, imparting fresh fruit aromas [10,11,12]. Currently, excessively high levels of ethanol and higher alcohols present a significant challenge for the industry [6,13,14]. Therefore, it is essential to implement effective regulatory measures to optimize the production of these compounds, thereby enhancing the overall flavor quality of wine.

Screening for low-alcohol yeast strains represents the most cost-effective biological strategy for reducing both ethanol and higher alcohol content in wine [15,16]. A primary approach involves redirecting sugar metabolism toward glycerol synthesis rather than ethanol production by regulating the glycerol pathway, thereby minimizing the formation of target alcohols. Glycerol synthesis in yeast is dependent on a single pathway that comprises two enzymatic reactions. Firstly, glycerol-3-phosphate dehydrogenase catalyzes the reduction in dihydroxyacetone phosphate (DHAP) to glycerol-3-phosphate. Subsequently, glycerol-3-phosphatase (GPP) catalyzes the dephosphorylation of glycerol-3-phosphate to produce glycerol [17]. The *GPD1* gene encodes glycerol-3-phosphate dehydrogenase, serving as a critical target for regulating glycerol synthesis. An increase in *GPD1* copy number accelerates glucose consumption, enhances glycerol yield, and significantly suppresses ethanol production [18,19,20]. Overexpression of *GPD1* in yeast lacking the pyruvate decarboxylase (PDC) gene enhances glycerol-3-phosphate dehydrogenase activity while mitigating side effects (e.g., acetate or acetaldehyde accumulation) that typically arise from overexpression in the original strain [21,22]. This strategy achieves a simultaneous reduction in ethanol production, an increase in glycerol yield, and better control of byproducts through targeted metabolic flux engineering, thereby providing essential technological support for the brewing of low-alcohol, high-quality wines.

Aquaporins, as specific transmembrane channels, play a crucial role in maintaining cellular water homeostasis by facilitating water transport across membranes [23]. This protein family includes not only pure water transporters but also subtypes that transport small polar molecules such as glycerol and larger alcohols. Their activity is subject to multi-level regulation: expression levels progressively alter membrane permeability, while regulatory mechanisms mediate rapid cellular responses to external stresses [24]. The crystal structure of the *AQY1* aquaporin in *S. cerevisiae* reveals that its N-terminal tyrosine residue (Tyr31) extends into the channel lumen, forming a physical block. Under hyperosmotic conditions that induce cellular dehydration, volume changes may trigger an osmotic sensing mechanism [25]. Notably, glycerol plays a critical protective role in osmotic regulation due to its highly hydrophilic hydroxyl group. Based on these mechanistic correlations, we hypothesize that alterations in intracellular glycerol concentration in *Saccharomyces cerevisiae* (*S. cerevisiae*) may induce osmotic stress by modulating aquaporin activity, ultimately affecting osmotic homeostasis and related metabolic processes. Accordingly, we designed experiments to investigate the effects of overexpressing relevant genes on alcohol and ester synthesis.

To elucidate the regulatory mechanisms of target genes influencing the fermentation characteristics of *S. cerevisiae*, this study integrated metabolic phenotyping with transcriptomic analysis. By constructing the engineered strains BY-*AQY1* and BY-*AQY1*/*GPD1*, we systematically monitored fermentation kinetics and metabolite profiles. RNA-sequencing (RNA-seq) was utilized to analyze global gene expression changes at the conclusion of fermentation, with the objective of providing a theoretical foundation for metabolic engineering aimed at optimizing yeast aroma production and minimizing fusel alcohols.

## 2. Materials and Methods

### 2.1. Yeast Strains

*S. cerevisiae* BY4741 (*MATa*, *his3Δ1*, *leu2Δ0*, *met15Δ0*, *ura3Δ0*, Laboratory storage) and *Escherichia coli* JM109 recipient cells (Takara, Beijing, China) and the pY26 plasmid (Laboratory storage) were used for constructing BY-*AQY1* and BY-*AQY1/GPD1* co-expressing strains. 

Yeast extract–peptone–dextrose (YPD) solid medium contained 20 g/L glucose, 20 g/L peptone, 10 g/L yeast extract, and 20 g/L agar powder.

Luria–Bertani (LB) medium contained 10 g/L NaCl, 10 g/L peptone, 5 g/L yeast extract, and 20 g/L agar powder.

Synthetic dextrose without uracil (SD-URA) medium contained 26.7 g/L Minimal SD Base, 1.29 g/L dropout supplement-URA, and 20 g/L agar powder.

The must used for seed culture and fermentation was prepared in accordance with the Triple M chemically defined must (CDM) [26] preparation method and sterilized by 0.22 µm filtration.

### 2.2. Construction of Overexpressing Strains

Primer design: Design 20 bp gene-specific amplification primers that target regions 300 bp upstream and downstream of the open reading frame (ORF) of the target gene. At the 5′ ends of both the forward and reverse primers, 20 bp arms homologous to the sequences flanking the polyclonal sites of the pY26/pY26-*AQY1* vector were incorporated. Consequently, the sequence of the forward primer consists of the “vector upstream homologous arm + gene-specific forward sequence,” whereas the reverse primer comprises the “vector downstream homologous arm + gene-specific reverse sequence.” Gene fragment amplification and purification: Target fragments were amplified by polymerase chain reaction (PCR) using genomic DNA from *S*. *cerevisiae* BY4741 as the template, along with the aforementioned primers. The PCR products were verified via agarose gel electrophoresis and then purified and recovered. Homologous recombination and plasmid construction: The purified gene fragment was mixed with the double-digested linearized vector pY26/pY26-*AQY1* in a molar ratio of 2:1 for fragment to vector. The mixture was incubated at 37 °C for 30 min using the ClonExpress II Homologous Recombination Kit to facilitate the assembly of the recombinant plasmids pY26-*AQY1* and pY26-*AQY1*/*GPD1*. Subsequently, it was transformed into BY4741 yeast along with the empty vector using the lithium acetate (LiAC) method. Transfected cells were cultured overnight at 30 °C in YPD medium, spread onto SD-Ura plates (30 °C, 2 d), and screened for transformants. DNA was extracted for subsequent confirmation.

### 2.3. LiAC Method for Yeast Conversion

Preparation of competent cells: Yeast strains were streaked on YPD plates and incubated for 2–3 d. A single colony was inoculated into 30 mL of YPD liquid medium and cultured for 12–16 h. This pre-culture was diluted in 50 mL of fresh YPD medium to an initial OD_600_ of 0.10 and grown at 30 °C with shaking (150 rpm) until the OD_600_ reached 0.40–0.60. Cells were harvested by centrifugation (4000× *g*, 5 min, 4 °C), washed once with 25 mL of ice-cold sterile water, and resuspended in 1 mL of 0.10 M LiAc. After a final centrifugation, the pellet was resuspended in 400 µL of 0.10 M LiAc to obtain competent cells.

Transformation procedure: Carrier ssDNA was denatured by boiling for 5 min and immediately chilled on ice. A transformation mixture was prepared by sequentially adding 240 μL of 50% PEG 3500, 36 μL of 1.0 M LiAc, 50 μL of denatured ssDNA (2.0 mg/mL), 34 μL of plasmid DNA (0.10–10 μg), and 50 μL of competent cell suspension into a 1.50 mL tube, followed by thorough vortexing after each addition. The mixture was incubated at 30 °C for 30 min, subjected to a heat shock at 42 °C for 40 min, and then centrifuged (8000× *g*, 15 s). The supernatant was discarded, and the cell pellet was resuspended in 800 μL of YPD medium. After recovery cultivation overnight at 30 °C with shaking, the cells were spread onto appropriate selective plates.

### 2.4. Fermentation Conditions

Yeast strains were inoculated onto SD-Ura solid medium and incubated at 30 °C for 48 h. Single colonies were subsequently picked and subcultured in 50 mL of simulated grape juice at 25 °C, with shaking at 150 rpm for 24 h. The activated culture was then inoculated at a concentration of 1 × 10^6^ cfu/mL into 150 mL conical flasks containing simulated grape juice, which had an initial sugar concentration of 200 g/L. Fermentation was carried out at 25 °C with shaking at 100 rpm, while monitoring CO_2_ loss every 24 h. Fermentation curves were plotted using time versus cumulative CO_2_ loss rates, and the fermentation process was continued until the residual sugar content reached 4 g/L or less, at which point the fermentation was considered complete. Three biological replicates were set up. All quantitative analysis indicators were sampled and tested following the completion of fermentation.

### 2.5. Measurement of Basic Physical and Chemical Parameters

High-performance liquid chromatography (HPLC) was used to determine the concentrations of residual sugars, glycerol, ethanol, and organic acids at the end of the fermentation. An HPX-87H hydrogen ion column (Bio-Rad Aminex, Hercules, CA, USA) (300 mm × 7.8 mm) equipped with a DAD UV and RID-type differential detector (ThermoFisher, Shanghai, China) was used as the chromatographic column. The mobile phase was a 5 mM H_2_SO_4_ (HPLC-grade) aqueous solution set at a flow rate of 0.60 mL/min. The column temperature was maintained at 60 °C, and the detection wavelengths were set at 210 nm and 254 nm. The final injection volume was 20 μL. Organic acid standards were prepared as 100 g/L tartaric acid, 100 g/L lactic acid, 500 g/L malic acid, 500 g/L citric acid, and 500 g/L pyruvic acid standard master-batches. Different amounts of the reserve solution were pipetted into 1 mL of the system to prepare mixed master-batches. Each mixed master-batch was subjected to gradient dilution, followed by injection into the liquid chromatograph (Kejie, Nanjing, China). Relevant organic acids were identified based on their respective peak retention times after 30 min of operation. Standard curves were generated using various concentrations of the standards as horizontal coordinates and the corresponding peak areas as vertical coordinates. Linear regression equations were derived from these curves.

Alcohol determination was performed using headspace solid-phase micro-extraction gas chromatography–mass spectrometry (GC-MS) (Agilent, Xi’an, China). Briefly, 5 mL of wine sample, 1 g of NaCl, and 10 μL of 4-methyl-2-pentanol (1.04 g/L) were added to a 20 mL headspace vial. The mixture was equilibrated at 40 °C for 10 min, extracted for 30 min, and then injected into the port for resolution for 8 min. A DVB/CAR/PDMS extraction fiber (50/30 μm coating thickness, 2 cm retractable length) and a SPME57330-U coupling handle (Supelco, Bellefonte, PA, USA) were used. The injection port was set at 230 °C for 5 min, and each sample was analyzed twice. For GC-MS, a DB-WAX polar column (60 × 0.25 mm × 0.25 mm) was used (Agilent, Xi’an, China). The temperatures of the ion source, connecting rod, and injection port were 200, 220, and 230 °C, respectively. The carrier gas was high-purity helium (≥99.999%) at a flow rate of 1.50 mL/min. The procedure involved holding the temperature at 40 °C for 5 min, then increasing it to 130 °C at 3 °C/min and to 250 °C at 4 °C/min. The temperature was then maintained at 250 °C for 5 min, resulting in a total running time of 60 min. The ion source was an electron ionization (EI) source at 70 eV voltage. Mass spectrometry was performed in the scanning range of 25–350 atomic mass units (amu) at a frequency of 50 Hz. The quantitative method determines concentration directly through standard curve fitting. For the preparation of the standard curve, a series of standard solutions of the target higher alcohol is prepared. An internal standard, either 4-methyl-2-pentanol or 2-octanol, is added at a fixed concentration to correct for sample injection errors. The standard samples are processed simultaneously according to the established sample pretreatment steps to create a standard curve that correlates peak area with concentration.

### 2.6. Transcriptomic Sample Preparation and Analysis

After fermentation is complete, 100 mL of fermentation broth was collected and centrifuged at 4000× *g* for 5 min at 4 °C. The supernatant was discarded, and the cell pellet was resuspended in 1 mL of sterile water. A second centrifugation step was performed at 8000× *g* for 1 min at 4 °C. The resulting sample was rapidly frozen in liquid nitrogen and subsequently stored at −80 °C prior to dispatch to Guangzhou Gedi’ao Company for RNA sequencing transcriptomic analysis. RNA extraction and library preparation were conducted following established protocols. Library quality assessment utilized both the Agilent 2100 Bioanalyzer and the ABI StepOnePlus qPCR system. Qualified libraries underwent quality control sequencing on the Illumina HiSeq 2000 platform. Raw data were filtered to yield clean reads, which were aligned using Bowtie2 for reference gene alignment and HISAT for reference genome alignment. Gene expression levels were normalized using fragments per kilobase of transcript per million mapped read (FPKM) values. Differentially expressed genes were identified employing the NOISeq algorithm with a threshold of |log_2_FC| ≥ 1 and probability ≥ 0.8. Significant differentially expressed gene sets were subjected to Gene Ontology (GO) enrichment analysis (corrected *p* ≤ 0.05) and Kyoto Encyclopedia of Genes and Genomes (KEGG) pathway analysis (Q-value ≤ 0.05).

### 2.7. Data Analysis

Data were preliminarily processed using SPSS 20.0 (SPSS Inc, Chicago, IL, USA). Data are expressed as the mean ± standard deviation. One-way ANOVA was performed to determine significant differences among the experimental and control groups. The confidence interval for one-way ANOVA was set at 95%. Heat maps and GO and KEGG analyses were performed using the OmicShare tools, a free online platform for data analysis (https://www.omicshare.com/tools/Home/Task, (accessed on 10 December 2025)). Figures were plotted using the Origin 2021 software (OriginLab,  Northampton, MA, USA).

## 3. Results

### 3.1. Growth State of Recombinant Strains

**Figure 1:**
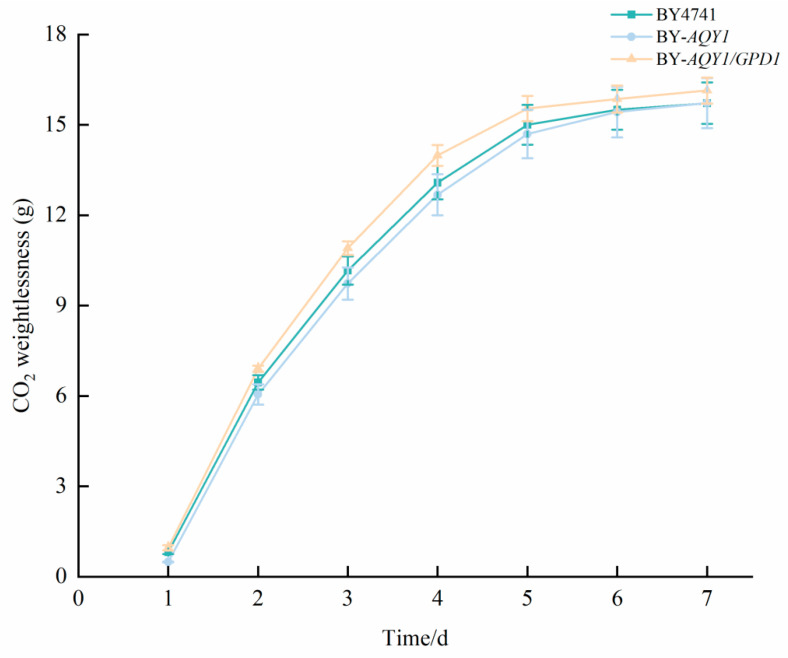
CO_2_ release of recombinant strains. Fermentation experiments were conducted at 5 °C and 100 rpm/min. Data represent the mean ± standard deviation from three independent replicate experiments.

During alcoholic fermentation, the release of gaseous carbon dioxide serves as an indicator of sugar consumption and the progression of fermentation kinetics. We interpret the CO_2_ release curve as a proxy for microbial metabolic activity. By analyzing the differences in the accumulated CO_2_ release curves among engineered strains, we can indirectly compare their overall metabolic intensity. An earlier and more robust CO_2_ release typically signifies faster substrate utilization and shorter fermentation cycles, which has favorable implications for improving the production efficiency of biotechnological processes. The results of the fermentation kinetics are presented in Figure 1. All strains completed fermentation within 7 days. Among these, strain BY-*AQY1* released 15.72 ± 0.83 g of CO_2_, which is slightly lower than the 15.72 ± 0.69 g released by strain BY4741; however, no significant difference in total weight loss was observed between the two strains by day 7. The CO_2_ release from the BY-*AQY1*/*GPD1* strain reached 16.14 ± 0.43 g, indicating a 2.67% increase compared to BY4741. These findings suggest that the overexpression of *AQY1* has a marginal inhibitory effect on the early fermentation rate but does not influence the overall fermentation cycle or the degree of fermentation. Conversely, while the co-expression of *AQY1* and *GPD1* resulted in an increased fermentation rate, it had only a limited effect on the fermentation cycle and the final degree of fermentation.

### 3.2. Basic Physicochemical Properties of Recombinant Strains

Upon completion of fermentation, the primary metabolites of each strain were measured and analyzed. As illustrated in Table 1, notable differences were observed among the strains with respect to glycerol, ethanol, flavor compounds, and organic acids. In terms of carbon metabolism, residual sugar levels remained relatively consistent across strains, ranging from 0.68 to 0.83 g/L. The ethanol yield of strain BY-*AQY1* increased by 2.25% compared to BY4741, accompanied by an improvement in ethanol productivity. In contrast, the BY-*AQY1*/*GPD1* strain demonstrated decreases of 6.32% in ethanol yield and 6.82% in productivity in comparison to BY4741. Regarding glycerol synthesis, BY-*AQY1*/*GPD1* exhibited the highest yield, which was 10.66% greater than that of BY4741, while strain BY-*AQY1* also showed a slight increase. With respect to flavor compound composition, the ester content in BY-*AQY1* was significantly higher than that in BY4741, increasing by 7.15%, while BY-*AQY1*/*GPD1* did not show a significant difference compared to BY4741. Additionally, BY-*AQY1* exhibited a 14.60% reduction in total higher alcohols compared to BY4741, whereas BY-*AQY1*/*GPD1* displayed no significant change. Organic acid analyses revealed that BY-*AQY1*/*GPD1* produced significantly more acetic acid than BY4741, with a 14.75% increase. Tartaric acid levels in BY-*AQY1* were slightly lower than those in BY4741, decreasing by 6.83%, while BY-*AQY1*/*GPD1* showed slightly higher levels than BY4741. No significant differences were noted among the strains for citric acid, pyruvic acid, malic acid, succinic acid, and lactic acid. In summary, the metabolite analysis indicates that compared to the control strain BY4741, the engineered strain BY-*AQY1* significantly increased ester production and reduced higher alcohol content while enhancing both ethanol yield and productivity. The *GPD1*-overexpressing strain BY-*AQY1*/*GPD1* exhibited markedly elevated glycerol and acetate production but reduced ethanol synthesis capacity. No significant differences were observed among the strains concerning residual sugar and most organic acid contents.

### 3.3. Volatile Compounds of Recombinant Strains

**Figure 2:**
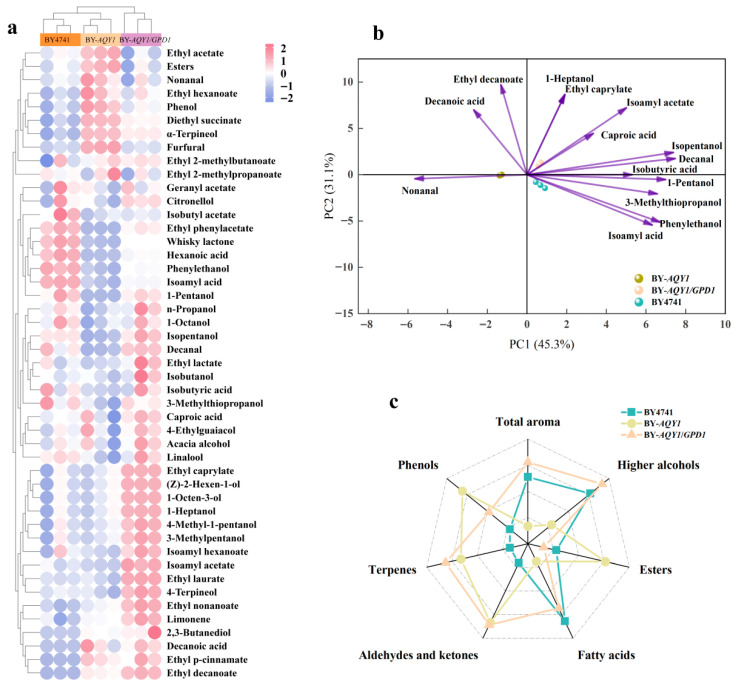
Analysis of volatile components in recombinant strains. Each strain fermentation is set up with three biological replicates. (**a**) Volatile compound heatmap. (**b**) Principal component analysis of volatile substances. (**c**) Proportion of volatile compounds (e.g., alcohols, esters, acids, aldehydes/ketones, terpenes, phenols).

To investigate the overall differences in volatile flavor compounds among various strains, this study performed a heatmap analysis based on the content of major volatile compounds (Figure 2a). The results indicate that BY-*AQY1*/*GPD1* forms a distinct cluster in the clustering diagram, exhibiting a unique flavor profile. In contrast, BY4741 and BY-*AQY1* cluster together, demonstrating a high similarity in the abundance of most flavor compounds. Notably, BY-*AQY1* exhibited significantly higher ethyl acetate content than both BY4741 and BY-*AQY1*/*GPD1*, with the total ester content being the highest among the three strains. Long-chain esters, such as ethyl decanoate and ethyl laurate, were most abundant in BY-*AQY1*/*GPD1*. Regarding the composition of higher alcohols, BY-*AQY1*/*GPD1* displayed significantly elevated levels of n-propanol, 4-methyl-1-pentanol, 3-methylpentanol, 1-heptanol, and 2,3-butanediol compared to BY4741 and BY-*AQY1*. Conversely, BY4741 exhibited the highest levels of isopentanol and 3-methylthiopropanol, while BY-*AQY1* showed significantly lower levels of phenylethanol compared to the other samples. Terpenoid analysis revealed that BY-*AQY1*/*GPD1* contained the highest levels of limonene and 4-terpineol, whereas BY-*AQY1* exhibited significantly higher α-terpineol content than the other samples, and BY4741 contained more linalool than BY-*AQY1*. This analysis suggests that BY-*AQY1*/*GPD1* exhibits a distinctive flavor profile resulting from its unique accumulation of long-chain esters, specific higher alcohols, and certain terpenes, thereby forming an independent cluster in the analysis. Although BY4741 and BY-*AQY1* are generally similar, variations in key flavor compounds such as ethyl acetate, phenylethanol, and α-terpineol contribute to subtle flavor distinctions between the two.

Principal component analysis (PCA) of volatile components with an odor activity value (OAV) greater than 1 (Figure 2b) revealed tight clustering among the samples, indicating strong data reproducibility. The cumulative variance explained by the first two principal components reached 76.4%. Compounds such as isoamyl acid, isopentanol, 1-pentanol, caproic acid, phenylethanol, and isoamyl acetate exhibited positive correlations with BY4741 and BY-*AQY1*/*GPD1*, while nonanal, phenethyl alcohol, and 3-methylthiopropanol displayed significant negative correlations with these strains. Additionally, both BY4741 and BY-*AQY1*/*GPD1* were positioned in the positive direction of principal component 1 (PC1), whereas the BY-*AQY1* strain consistently appeared in the negative direction of PC1.

To comprehensively evaluate the flavor profiles of various samples, this study selected six key flavor compounds—higher alcohols, esters, fatty acids, aldehydes and ketones, terpenes, and phenols—as indicators. A radar chart was created for visual analysis (Figure 2c). The results demonstrated noticeable differences in the radar chart patterns among the three samples, indicating unique characteristics in their overall composition and distribution of flavor compounds. The concentration range of total volatile components was 273.25–322.12 mg/L. The proportions of alcohols in BY4741, BY-*AQY1*, and BY-*AQY1*/*GPD1* were 84.15%, 81.73%, and 84.79%, respectively, while the proportions of esters were 8.72%, 10.63%, and 8.26%. BY4741 exhibited moderate levels of higher alcohols, fatty acids, and phenolic compounds, but relatively lower levels of esters and terpenoids. BY-*AQY1* displayed the highest levels of esters, aldehydes/ketones, and terpenoids among the three strains; however, its higher alcohol content was significantly lower than that of BY4741 and BY-*AQY1*/*GPD1*. In contrast, BY-*AQY1*/*GPD1* presented a distinctly different profile, with significantly higher levels of higher alcohols and maintaining relatively high levels of terpenoids, while its fatty acid content was comparable to that of BY4741.

### 3.4. Transcriptomic Analysis

**Figure 3:**
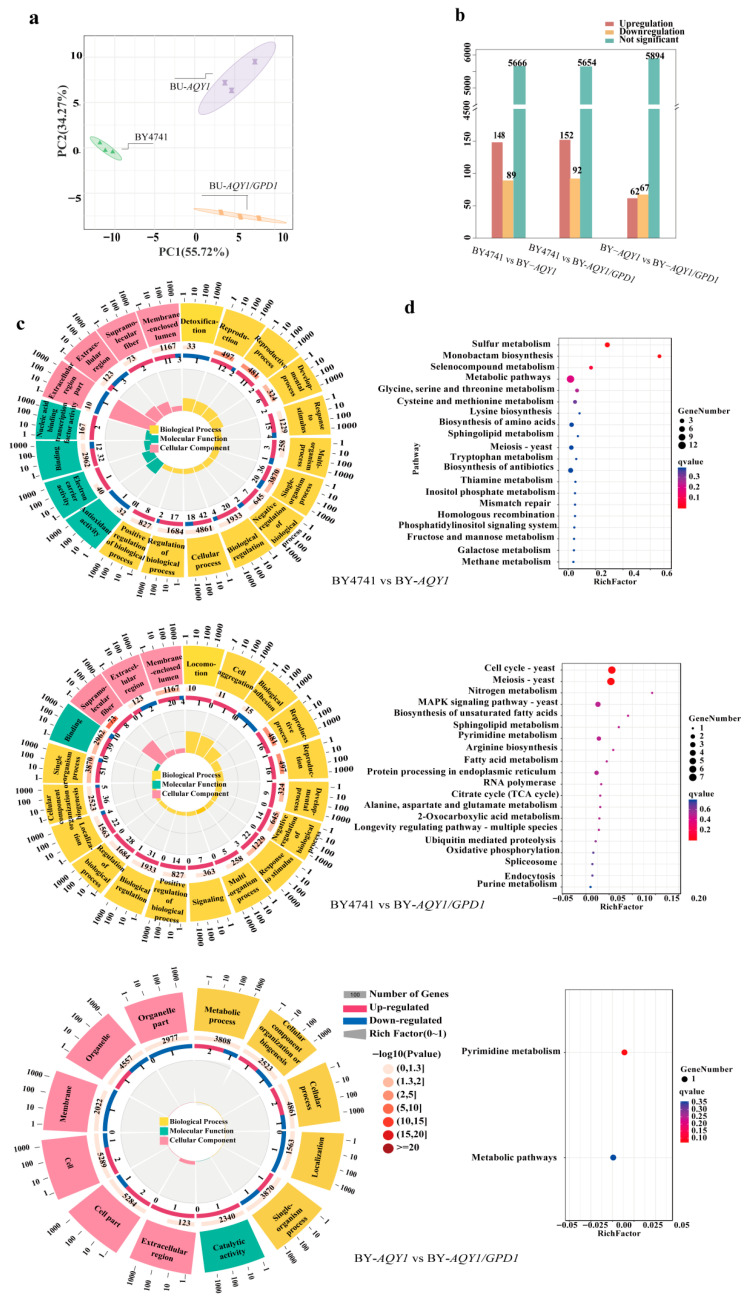
Transcriptome data analysis results. Each strain fermentation is set up with three biological replicates. (**a**) Principal component analysis (PCA). (**b**) Differentially expressed gene analysis. (**c**) GO functional enrichment analysis. First Circle: Enriched categories, with the gene count scale outside the circle. Different colors represent distinct categories; Second Circle: Number of genes in this category among background genes and their P-values. Longer bars indicate more genes, while redder colors signify smaller values; Third Ring: Bar chart showing the proportion of up-regulated and down-regulated genes. Fourth Ring: RichFactor values for each category (calculated as the number of foreground genes in that category divided by the number of background genes). Each small grid on the background grid line represents 0.1. (**d**) KEGG pathway enrichment analysis.

Transcriptome sequencing of the fermentation broth from the recombinant strain generated an average of 37,789,244 raw reads. Following quality control filtering, a total of 37,518,619 clean reads were obtained, representing over 99.10% of the total, with more than 89.71% mapped to the exonic regions of the reference genome (with introns and intergenic regions accounting for less than 10.29%), indicating excellent data quality. The results of principal component analysis (PCA) (Figure 3a) revealed significant transcriptional differences among the strains, while the biological replicates within each strain exhibited high clustering, demonstrating robust experimental reproducibility. A pairwise comparison to identify differentially expressed genes (Figure 3b) indicated that, compared to BY4741, 148 genes were significantly upregulated and 89 genes were significantly downregulated in BY-*AQY1*, while in BY-*AQY1*/*GPD1*, 152 genes were upregulated and 92 genes were downregulated. Further comparisons between BY-*AQY1* and BY-*AQY1*/*GPD1* revealed relatively minor transcriptomic differences, identifying 62 genes that were upregulated and 67 genes that were downregulated. These results suggest that genetic engineering induces systematic changes at the transcriptional level, with the gene sets affected by different modification strategies exhibiting both overlapping and unique characteristics. This provides essential insights for elucidating the regulatory mechanisms underlying differences in metabolic phenotypes.

Gene Ontology (GO) functional enrichment analysis of differentially expressed genes revealed notable similarities and differences in enriched functional categories across the comparison groups (Figure 3c). In the comparison between BY4741 and BY-*AQY1*, differentially regulated genes showed significant enrichment in cellular processes, signaling pathways, and developmental regulation. This finding suggests that the modification of the BY-*AQY1* strain may have affected the regulatory networks that govern cellular interactions and environmental responses. In the comparison between BY4741 and BY-*AQY1*/*GPD1*, differentially expressed genes were enriched in biological processes, including reproduction, development, and stress response, as well as in detoxification and antioxidant functions. Additionally, enrichment in electron carrier activity and nucleic acid-binding transcription factor activity indicates alterations in redox metabolism and transcriptional regulation within this strain. These changes are likely closely associated with shifts in the redox coenzyme balance induced by *GPD1* overexpression. In the direct comparison of the two engineered strains, BY-*AQY1* and BY-*AQY1*/*GPD1*, differentially expressed genes were primarily enriched in functions related to basic metabolism and cellular structure. This observation indicates that the introduction of *GPD1* predominantly influenced core material metabolism and the construction of cellular structures within the BY-*AQY1* background, instead of significantly affecting developmental processes or signaling regulation.

KEGG pathway enrichment analysis (Figure 3d) indicates that, in the comparison of BY4741 and BY-*AQY1*, enriched pathways are predominantly associated with sulfur metabolism and various amino acid biosynthesis, while also encompassing genetic processes such as meiosis, mismatch repair, and homologous recombination. This finding suggests that *AQY1* may influence the basal metabolism and genetic stability of yeast. The comparison between BY4741 and BY-*AQY1*/*GPD1* revealed significant enrichment in pathways related to the cell cycle, mitosis, MAPK signaling, RNA polymerase, spliceosome, and endocytosis, which are important for cell proliferation and regulation. It also highlighted energy and material metabolism processes, including the TCA cycle, oxidative phosphorylation, fatty acid metabolism, and nitrogen metabolism. This implies that the introduction of *GPD1* may enhance the regulation of the cellular cycle and energy metabolism functions. In contrast, the comparison of BY-*AQY1* and BY-*AQY1*/*GPD1* identified significant enrichment, exclusively in pyrimidine metabolism and metabolic pathways. This suggests that while the two strains exhibit similar profiles across most pathways, *GPD1* overexpression primarily impacts nucleotide metabolism and related fundamental metabolic networks.

#### 3.4.1. Effect of Glycerol Metabolism on Higher Alcohols

**Figure 4:**
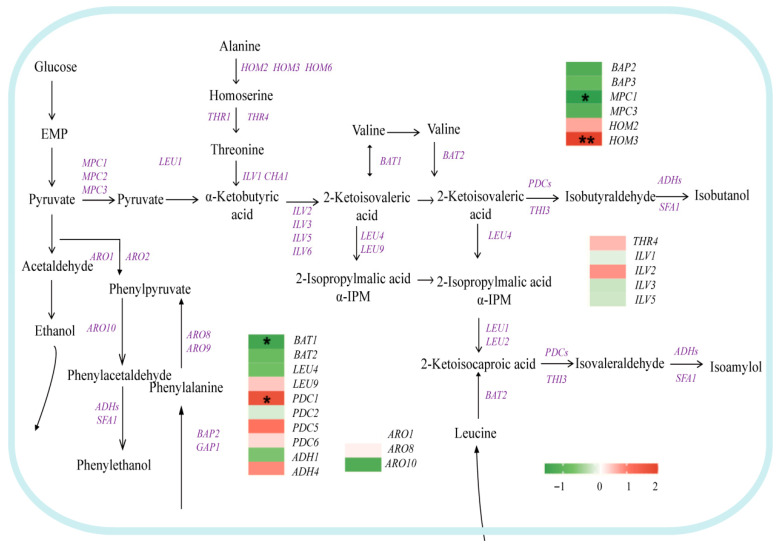
Transcriptional changes in genes related to higher alcohol metabolism pathway (BY4741 vs. BY-*AQY1*). Purple indicates genes involved in this process. * denotes genes with |log_2_FC| ≥ 1 and *p* < 0.05; ** denotes genes with |log_2_FC| ≥ 1 and *p* < 0.01.

Higher alcohols can be categorized, based on the origin of α-keto acids, into two pathways: the Ehrlich pathway and the Harris pathway. The Ehrlich pathway involves the genes *BAP2/3* and *BAT2*, which encode proteins responsible for branched-chain amino acid metabolism. *BAP2/3* is localized in the mitochondria, while *BAT2* catalyzes the synthesis of α-keto acids in the cytoplasm by converting amino acids into α-keto acids. These α-keto acids are subsequently transformed into higher alcohols through decarboxylation and dehydrogenation [27,28]. A knockdown of either *BAP2/3* or *BAT2* significantly reduces the production of higher alcohols [28]. In this study, the simultaneous downregulation of both *BAP2/3* and *BAT2* in BY-*AQY1* confirmed that the suppression of their expression is a fundamental mechanism affecting higher alcohol production. Additionally, downregulation of *LEU4*, which encodes α-isopropylmalate synthase, inhibits the conversion of α-ketoisovaleric acid to isopentanol. The downregulation of *BAP2/3*, which are branched-chain amino acid transporter genes, decreases the transport of leucine, valine, and phenylalanine, further reducing the production of isopentanol and phenethyl alcohol [29,30]. Collectively, *AQY1* overexpression reshapes the network of higher alcohol synthesis by inhibiting genes related to amino acid metabolism (Figure 4).

#### 3.4.2. Effect of Glycerol Metabolism on Ethanol

**Figure 5:**
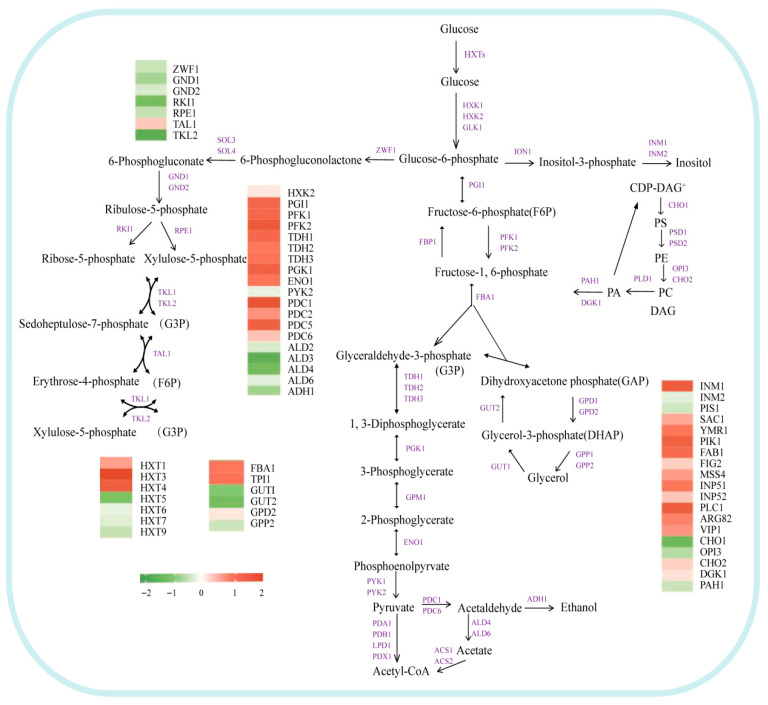
Transcriptional changes in genes related to carbon metabolism pathways (BY4741 vs. BY-*AQY1*/*GPD1*). Purple indicates genes involved in this process.

Glucose-6-phosphate serves as a common precursor for both glycerol and ethanol, experiencing intricate metabolic flux regulation through glycolysis (Embden–Meyerhof–Parnas, EMP), gluconeogenesis, and the pentose phosphate pathway (PPP) [31,32]. The upregulation of fructose-1,6-bisphosphate kinase genes *PFK1* and *PFK2* in co-expressing strains not only directly facilitates the accumulation of precursors for glycerol synthesis but may also inhibit the activity of genes associated with ethanol synthesis, consequently reducing ethanol yield (Figure 5). This conclusion is further supported by the observed increase in ethanol production in *PFK*-deficient strains [33,34]. Simultaneously, the downregulation of several genes within the pentose phosphate pathway (PPP)—including *ZWF1*, *GND1/2*, *RKI1*, and *TKL2*—correlates with the decrease in ethanol yield that was observed [35,36,37,38,39].

Enhanced glycerol metabolism plays a critical role in reducing ethanol yield. The upregulation of the fructose bisphosphate aldolase gene *FBA1* leads to the accumulation of its products, glycerol-3-phosphate and dihydroxyacetone phosphate [40,41]. Dihydroxyacetone phosphate serves as the precursor for glycerol synthesis, undergoing conversion into glycerol through the action of significantly upregulated glycerol-3-phosphate dehydrogenase genes (*GPD1*, possibly along with *GPD2*), along with the upregulation of the glycerol transporter gene *FPS1* [42,43]. Conversely, the downregulation of several aldehyde dehydrogenase genes (*ALD2*, *ALD3*, *ALD4*, and *ALD6*) inhibits the conversion of acetaldehyde to acetate [44,45], while the reduced expression of the alcohol dehydrogenase gene *ADH1* directly impairs the final step of converting acetaldehyde to ethanol, collectively resulting in a significant decrease in ethanol production [46].

#### 3.4.3. Effect of Glycerol Metabolism on Esters

**Figure 6:**
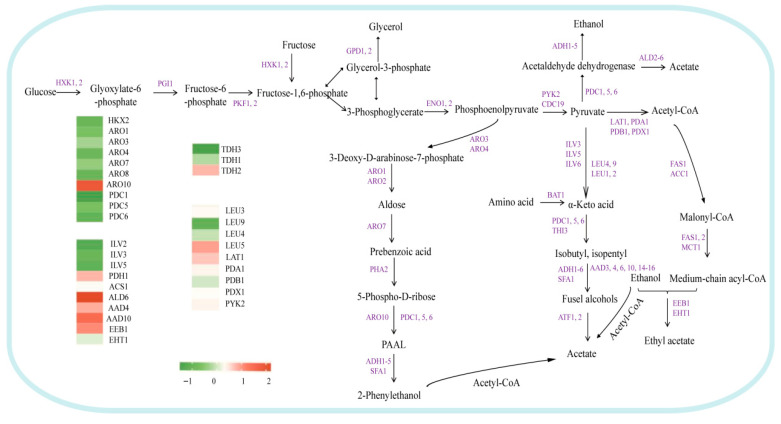
Transcriptional changes in genes related to ester metabolism (BY-*AQY1* vs. BY-*AQY1*/*GPD1*). Purple indicates genes involved in this process.

Ethyl esters and acetate salts are essential flavor compounds that contribute to the fruitiness of wine. In yeast, the synthesis of ethyl esters is primarily regulated by the ethanol O-acyltransferase genes *EHT1* and *EEB1* (Figure 6). Research indicates that modulating the expression of these two genes can effectively alter ester content [47,48]. Ethyl acetate, a significant flavor compound synthesized from acetyl-CoA and ethanol, exhibits concentration regulation based on substrate levels [49,50]. Cytoplasmic acetyl-CoA is predominantly generated through the *PDH* bypass pathway [51,52], which involves genes such as *PDH1* (pyruvate decarboxylase), *ALD6* (aldehyde to acetate), and *ACS1* (acetate to acetyl-CoA) [50,51,53,54]. In the BY-*AQY1*/*GPD1* strain, although key genes associated with the ester synthesis pathway—*PDH1*, *ALD6*, *ACS1*, and *EEB1*—were generally upregulated (with the exception of *EHT1*), the total ester content was significantly lower than that of the BY-*AQY1* strain, with ethyl acetate content reduced by 11.07%. Simultaneously, an increase in higher alcohol content was observed. This finding suggests that despite the enhanced expression of ester synthesis-related genes, the co-expression of *GPD1* and *AQY1* may have ultimately decreased ester yield by impacting the efficiency of precursor production (e.g., acetyl-CoA) or inducing a redistribution of carbon metabolic flux (e.g., toward glycerol and higher alcohols).

## 4. Discussion

Primary metabolites are essential substances synthesized by yeast to support fundamental life processes, including growth, reproduction, and metabolism. These metabolites encompass carbohydrates (e.g., glycerol, ethanol), organic acids, amino acids, higher alcohols, and esters, which are the focus of this study. In contrast, secondary metabolites are non-essential compounds produced during the stationary phase of microbial growth. These compounds are not directly related to basic growth and metabolism and were not detected in the metabolomic profile of this study. During the fermentation of *S. cerevisiae*, the synthesis of all major metabolites involved constitutes a continuous and interconnected process. Central to this process is glycolysis, which serves as the core hub for carbon flux. Here, extracellular glucose is transported into yeast cells and converted to pyruvate via glycolysis. Pyruvate acts as the primary precursor for all downstream major metabolites, specifically following these pathways: glycolysis → pyruvate → glycerol/ethanol synthesis; glycolysis → pyruvate → amino acids → higher alcohol/ester synthesis.

By engineering yeast strains co-expressing the aquaporin gene *AQY1* and the key glycerol synthesis gene *GPD1*, we observed significant alterations in glycerol and higher alcohol concentrations in the fermentation broth. However, these strains did not exhibit any substantial effects on fermentation time or rate. This finding suggests that the overexpression of *AQY1* and *GPD1* may not directly influence cell growth or the primary fermentation capacity. This lack of direct impact might be attributed to the primary roles of these genes in secondary metabolic pathways, such as glycerol synthesis, which exert limited influence on the principal pathways of glycolysis and ethanol fermentation [55]. Conversely, these genes may have activated intracellular feedback regulatory mechanisms that sustained stable fermentation rates through compensatory adjustments involving other genes or metabolic pathways [56].

Glycerol is a vital metabolic product in *S*. *cerevisiae*. Under hyperosmotic conditions, yeast synthesizes and accumulates glycerol to equilibrate intracellular and extracellular osmotic pressure, thereby preventing cellular dehydration [57]. *AQY1* is an essential protein that mediates the transmembrane transport of water and small molecules, including glycerol [58]. It collaborates with the glycerol channel Fps1p to regulate glycerol homeostasis in response to osmotic stress [59]. In hyperosmotic environments, yeast activates the HOG pathway, leading to the upregulation of *AQY1* and *GPD1* gene expression to facilitate glycerol accumulation. Additionally, *AQY1* indirectly influences cellular redox status by regulating glycerol transport efficiency [60]. The knockout of *AQY1* diminishes glycerol efflux and disrupts osmotic regulation under hypertonic conditions. While the overexpression of *AQY1* enhances the glycerol transport rate, if not co-regulated with Fps1p it may result in intracellular glycerol loss [61]. Overexpression of *AQY1* alone can accelerate glycerol loss; however, co-expression with *GPD1* yields synergistic effects. *GPD1* promotes glycerol synthesis, while *AQY1* reduces its efflux, collectively enhancing intracellular glycerol retention levels. This interaction explains the observed significant increase in glycerol content.

Overexpression of *AQY1* results in a significant reduction in higher alcohol levels. This phenomenon may occur because carbon sources, such as glucose, are redirected to the glycerol synthesis pathway, thereby decreasing the metabolic flux entering the Ehrlich pathway, which is responsible for higher alcohol production. Pyruvate and NADH generated through glycolysis are preferentially directed toward glycerol synthesis rather than being converted into acetyl-CoA, which is essential for the Ehrlich pathway [62]. Concurrently, Gpd1p catalyzes glycerol synthesis, consuming considerable amounts of NADH. The overexpression of *AQY1* may further exacerbate this consumption, leading to decreased intracellular NADH levels. Significant NADH consumption indicates a potential alteration in the intracellular redox state (NADH/NAD^+^ ratio). We hypothesize that this may lead to a reduction in the metabolic flux of pyruvate toward acetyl-CoA and the TCA cycle. However, this inference necessitates confirmation through direct detection of NADH/NAD^+^ ratios and enzyme activity assays. Since NADH serves as the primary reductant for the conversion of ketoacids into higher alcohols in the Ehrlich pathway [60], its depletion directly inhibits this process. Additionally, *AQY1* overexpression is associated with the downregulation of genes linked to the osmotic stress response pathway (e.g., *BAT1*/*2*, *LEU4*) [56], suggesting that the cells may be in a physiological state resembling osmotic adaptation or that this pathway may be activated. Further investigation is required to determine the specific degree of activation and its physiological implications.

The co-expression of the *AQY1* and *GPD1* genes significantly reduced ethanol production. Overexpression of *GPD1* enhanced the metabolic flux within the glycerol synthesis pathway, while the overexpression of *AQY1* inhibited glycerol efflux, thereby synergistically promoting intracellular glycerol accumulation [58]. This resource reallocation diverted substantial amounts of glycolytic intermediates, such as dihydroxyacetone phosphate (DHAP), along with the cofactor NADH, toward glycerol synthesis. Consequently, the activity of key enzymes responsible for converting acetaldehyde to ethanol—specifically, the aldehyde dehydrogenases *ALD2*, *ALD3*, *ALD4*, and *ALD6*—was inhibited [62], ultimately reducing the conversion of pyruvate to ethanol [63]. Simultaneously, the overexpression of *GPD1* further depletes NADH availability through its consumption, thereby inhibiting the activity of alcohol dehydrogenase (*ADH1*). We hypothesize that these factors collaboratively diminish ethanol synthesis [60].

Compared to the BY-*AQY1* strain, the BY-*AQY1*/*GPD1* strain exhibited significantly elevated levels of higher alcohols and markedly reduced ester compounds. The overexpression of *GPD1* substantially consumes the glycolytic intermediate dihydroxyacetone phosphate (DHAP) while enhancing glycerol synthesis, potentially accelerating glycolysis through metabolic feedback mechanisms, such as the activation of phosphofructokinase, and increasing pyruvate yield. Accumulated pyruvate enters the Ehrlich pathway through the replenishment reaction, promoting the synthesis of branched-chain amino acids and increasing the supply of higher alcohol precursors [64]. Simultaneously, excessive NADH consumption by *GPD1* may drive cells to regenerate NAD^+^ via ethanol or higher alcohol synthesis, thereby activating the reduction phase of the Ehrlich pathway. The reduction in ester production is attributed to the impaired conversion of pyruvate to acetyl-CoA, a key precursor for ester synthesis, resulting from the increased flux in glycerol synthesis [65]. Additionally, the activation of the glycerol pathway may suppress the expression of ester synthesis genes, such as *EHT1*, via the Snf1/Mig1 signaling pathway, collectively diminishing ester yield [66].

In actual sensory experiences, the perceptibility of volatile compounds, derived from variations in fermentation metabolites, is dependent on their concentration and odor activity value (OAV). An OAV of ≥1 indicates that a compound is detectable by human senses, with higher OAVs reflecting stronger sensory contributions. In this study, significant changes in the concentrations of key odor-active volatile compounds in the *AQY1* single-overexpressing strain and the *AQY1*/*GPD1* co-overexpressing strain resulted in pronounced OAV differences compared to the control group. Specifically, alterations in typical higher alcohols (e.g., amyl alcohol, isobutanol) and characteristic esters (primarily fruity and floral compounds) in the engineered strains exhibited OAVs substantially exceeding 1. This suggests that these concentration differences are not only chemically detectable but also possess practical sensory relevance, directly influencing the olfactory and gustatory characteristics of the fermented products. Conversely, minor fluctuations in certain volatile compounds with OAV < 1 in the engineered strains were identified, indicating that these subtle variations have negligible sensory impact and can be disregarded in practical sensory evaluations. Furthermore, the glycerol content in the engineered strain—a key textural component in wine—demonstrated that moderate increases enhance the smoothness of fermented products without adding greasiness. Together with volatile compounds, these elements collectively shape the overall perceptible sensory profile of the fermented product.

Combining metabolite detection and transcriptomic analysis, we elucidate the potential industrial benefits of the observed metabolic differences in fermentation production. These benefits are primarily manifested in two key areas: (1) Flavor Quality Optimization: Modulating the synthesis of key volatile compounds—such as decreasing higher alcohol content that contributes to undesirable odors while promoting the accumulation of esters with pleasant fruity aromas in engineered strains—can significantly enhance the sensory acceptability of fermentation products, thereby addressing a fundamental requirement for flavor control in industrial fermentation. (2) Enhanced Fermentation Quality and Product Stability: Moderately increasing glycerol content in engineered strains improves osmotic tolerance of yeast cells during fermentation, reduces cell mortality, and enhances process stability. Additionally, fine-tuning sugar and organic acid metabolism prevents the excessive accumulation of undesirable metabolites, ensuring consistent quality in large-scale industrial fermentation products. Furthermore, transcriptomic data reveal the molecular pathways that underlie metabolic regulation in *AQY1* and *AQY1*/*GPD1* strains. This knowledge hypothesis provides a theoretical basis for the rational modification of yeast strains in industrial fermentation and establishes a foundation for the practical application of these engineered strains in industrial production.

The limitations of this experiment are as follows: due to the absence of a control group overexpressing *GPD1* alone, this study is unable to fully isolate the contribution of *AQY1* from the baseline effects of *GPD1*, nor can it accurately determine whether their effects are purely additive or exhibit synergistic interactions. To address this limitation, our subsequent research will construct *GPD1* overexpression strains for complementation experiments. This approach will facilitate quantitative differentiation between the respective contributions of *AQY1* and *GPD1*, enabling more precise validation of their synergistic interaction mechanism.

## 5. Conclusions

This study developed engineered strains that regulate glycerol metabolism. The BY-*AQY1* strain achieved a synergistic increase in ethanol and flavor ester production while significantly reducing the levels of higher alcohols. In contrast, the BY-*AQY1*/*GPD1* strain exhibited a distinct high-glycerol-yield phenotype. These findings elucidate the specific regulation of yeast fermentation metabolic networks through various genetic modification strategies, providing a theoretical foundation for the molecular breeding of industrial yeast strains. This study undertook an integrated analysis of phenotypic and transcriptomic data, developing a mechanistic hypothesis for the observed phenomenon based on the correlations between the two datasets. This hypothesis necessitates additional validation through experiments, including enzyme activity assays and metabolic flux analysis. Following this, we will perform fermentation using actual grape substrates to assess the strain’s performance and its influence on product flavor.

## Figures and Tables

**Table 1:** Basic physicochemical properties of recombinant strains.

Overexpression Strain	BY4741	BY-*AQY1*	BY-*AQY1/GPD1*
Residual sugar (g/L)	0.68 ± 0.01 ^a^	0.83 ± 0.12 ^a^	0.70 ± 0.03 ^a^
Glycerol (g/L)	4.41 ± 0.24 ^c^	4.70 ± 0.04 ^a,b^	4.88 ± 0.12 ^a^
Ethanol (g/L)	86.73 ± 0.19 ^a,b^	88.68 ± 0.14 ^a^	81.25 ± 0.26 ^c^
Ethanol yield (g/g)	0.44 ± 0.00 ^a,b^	0.45 ± 0.00 ^a^	0.41 ± 0.00 ^c^
Esters (mg/L)	27.12 ± 0.50 ^b^	29.06 ± 0.35 ^a^	26.62 ± 1.23 ^b^
Total higher alcohols (mg/L)	261.51 ± 1.22 ^a^	223.32 ± 2.79 ^b^	273.12 ± 13.68 ^a^
Citric (g/L)	1.17 ± 0.08 ^a^	1.19 ± 0.06 ^a^	1.21 ± 0.06 ^a^
Tartaric (g/L)	1.61 ± 0.04 ^a,b^	1.50 ± 0.06 ^b^	1.62 ± 0.02 ^a^
Pyruvic (g/L)	0.30 ± 0.01 ^a^	0.31 ± 0.00 ^a^	0.30 ± 0.01 ^a^
Malic (g/L)	0.10 ± 0.00 ^a,b^	0.12 ± 0.01 ^a^	0.11 ± 0.01 ^a^
Succinic (g/L)	0.61 ± 0.00 ^b^	0.62 ± 0.04 ^b^	0.54 ± 0.07 ^b^
Lactic (g/L)	0.09 ± 0.01 ^b^	0.10 ± 0.02 ^a,b^	0.09 ± 0.01 ^b^
Acetate (g/L)	1.22 ± 0.04 ^b^	1.13 ± 0.01 ^b^	1.40 ± 0.07 ^a^

Notes: Different lowercase letters represent the significance of the difference (Duncan, *p* < 0.05). Data represent the mean ± standard deviation from three independent replicate experiments.

## Data Availability

The original contributions presented in this study are included in the article. Further inquiries can be directed to the corresponding authors.
